# Use of the Roadmap positive activity app in daily life among dementia caregivers: evidence of feasibility and acceptability

**DOI:** 10.3389/frdem.2026.1861809

**Published:** 2026-09-23

**Authors:** Kira S. Birditt, Yee To Ng, Lizbeth Benson, Allison Doroshewitz, Angela Turkelson, Noelle E. Carlozzi, Sung Won Choi

**Affiliations:** 1 Institute for Social Research, University of Michigan, Ann Arbor, MI, United States; 2 School of Medicine, University of Michigan, Ann Arbor, MI, United States

**Keywords:** acceptability, caregiving, feasibility, mHealth app, positive activities, well-being

## Abstract

**Background:**

Dementia caregivers often report poor mental health, which has implications for the quality of care, as well as care recipient well-being. Thus, it is imperative to identify modifiable factors that influence caregiver mental health and can be targeted through personalized interventions.

**Objective:**

The purpose of the present study was to examine the feasibility and acceptability of the Roadmap mHealth app, a positive activity intervention for dementia caregivers, and preliminary associations with caregiver mental health.

**Methods:**

A total of 20 dementia caregivers participated in a 14-day study, during which they received prompts to engage in Roadmap positive activities twice a day and complete nightly mobile surveys regarding their own and their care recipient’s mental health. Both caregivers and their recipients were asked to wear Fitbit® devices. Caregivers completed a feasibility and acceptability survey at the end of the study. Data were analyzed using descriptive statistics and 2-level multilevel linear models with days nested in participants.

**Results:**

The app showed high feasibility, with caregivers engaging in Roadmap positive activities on 87% of the days and completing 95% of nightly surveys. All caregivers and 75% of their recipients wore Fitbits®. The Fitbit® and nightly surveys were acceptable (above 80% agreement), but there were some aspects of the app that may warrant improvement (72–78% agreement). Same-day positive activities were associated with lower caregiver depression, lower appraisals of caregiving as stressful, and higher caregiver positive mood.

**Conclusion:**

Overall, the Roadmap app is feasible and shows promising associations with caregiver mental health, but some improvements are needed to improve acceptability. A larger, fully powered randomized controlled trial is needed to evaluate whether the Roadmap app improves caregiver mental health.

## Introduction

Roughly 11 million Americans provide unpaid care to people living with Alzheimer’s Disease and Related Dementias (ADRD) (Alzheimer’s Association, 2025). Unpaid caregivers are becoming increasingly vital in the long-term care of this vulnerable population (Ali et al., 2022; Freedman et al., 2022). Caregivers of people living with ADRD (care recipients) provide more hours of care, more hands-on and demanding caregiving, perform more medical and nursing tasks for which they have little or no training, and report greater caregiver burden and worse mental health compared to non-ADRD caregivers (Anderson et al., 2013; Kasper et al., 2015; Capistrant, 2016; Lee et al., 2019; Ali et al., 2022; Freedman et al., 2022). As care responsibilities accumulate, caregivers face their own health risks, including anxiety, burden, sleep problems, decreased physical activity, and chronic illness (Anderson et al., 2013; Kasper et al., 2015; Lee et al., 2019). These risks, in turn, may harm the mental health of care recipients (Capistrant, 2016). Addressing the needs of at-risk ADRD caregivers and their care recipients is an urgent public health priority (Cella et al., 2007). Thus, it is imperative to identify modifiable factors that influence caregiver mental health and can be targeted through personalized interventions.

Engaging in positive activities is associated with improved affect, as well as mental and physical well-being among ADRD caregivers (Mausbach et al., 2017). Overall, caregivers who engage in fewer pleasant activities report greater depression, higher negative affect, lower positive affect, less positive coping, more negative coping, and more sleep disturbance (Chattillion et al., 2013). Intensive ecological momentary assessment (EMA) studies have also shown within-person associations between leisure activities and improvements in caregiver affect, with some showing improvements in only positive affect and others showing improvements in both positive and negative affect (Cheng et al., 2022). Leisure activities also tend to be more effective when the activities are perceived as more pleasant, when caregivers report greater burden, and when caregivers engage in a variety of activities (Poon, 2022; Hwang and Kim, 2024).

To understand the links between caregiver engagement in positive activities and mental health outcomes among ADRD caregivers and care recipients, we developed the Positive Activities and Daily Mental and Physical Health among ADRD Caregiver-Care Recipient Dyads Model, which suggests that engaging in Roadmap positive activities will lead to improvements in ADRD caregiver mental health via, improvements in caregiver mood and better quality of interactions (as less stressful and more rewarding) between the caregiver and care recipient (Figure 1). These positive activities and improvements in caregiver mental health may also lead to improvements in care recipients’ mental health. The model incorporates aspects of several leading theories in the literature, including Pearlin’s stress process model of caregiving, behavioral activation theory, and dyadic illness management theory (Lewinsohn, 1974; Kanter et al., 2010; Lyons and Lee, 2018; Miller et al., 2019). This study examines associations between engaging in positive activities, caregiver mental health, and the potential mechanisms of action (positive and negative mood, appraisals of caregiving as stressful and rewarding). We also consider caregiver proxy reports of care recipient mental health to explore whether there are associations between caregiver positive activities and care recipient mental health.

**Figure 1:**
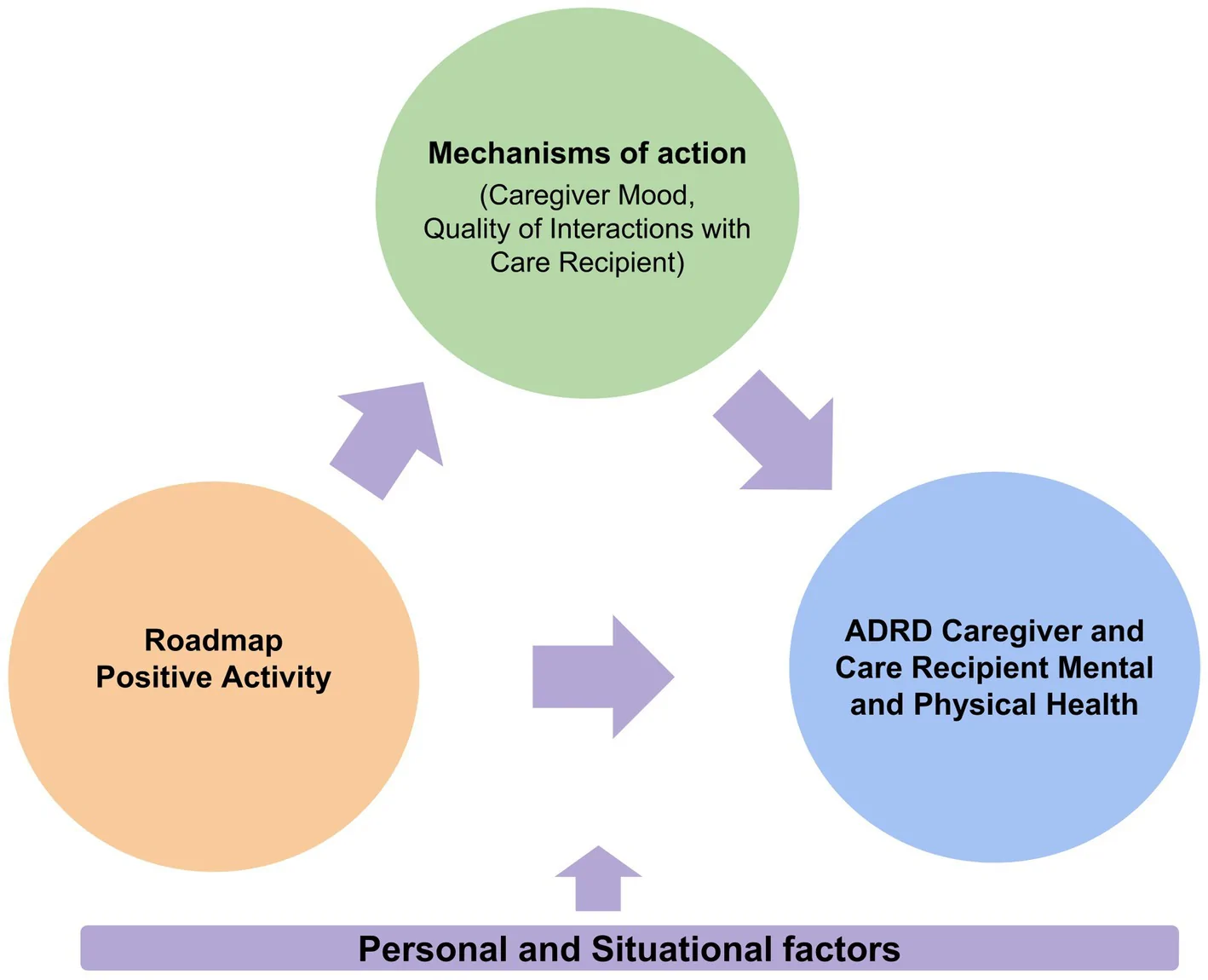
Positive activities and daily mental and physical health among ADRD caregiver-care recipient dyads model.

Existing positive psychology interventions demonstrate that increasing positive activities is beneficial for both the general population and dementia caregivers (Seligman et al., 2005; Sin and Lyubomirsky, 2009). A review of the literature on ADRD caregiver interventions found that cognitive behavioral therapy, which focuses on increasing positive activities and reducing negative thoughts, is more effective than other types of interventions (psychoeducational, mindfulness) (Sin and Lyubomirsky, 2009). For example, when compared to psychoeducation, dementia caregivers who also received pleasant activity scheduling reported decreased depression over 4 months (Lee et al., 2020). LEAF (Life Enhancing Activities for Frontotemporal dementia family caregivers) intervention, which includes journaling about positive events and gratitude, mindfulness, and acts of kindness, was associated with increased positive affect, reduced negative affect, reduced caregiver burden, and stress over a 5- or 6-week period (Au et al., 2015). These are all fairly high-burden interventions (e.g., include several sessions over a long period of time), and as such, there is an urgent need for low-burden, scalable interventions for improving mental health among caregivers.

One promising low-burden and scalable approach involves mobile health (mHealth) interventions, which leverage mobile apps to support individuals in their daily lives. Apps may be particularly well-suited for caregivers given their responsibilities and limited time. Lin et al. (2026) reviewed app-based interventions for family caregivers, including web-, mobile-, and tablet-based apps. Across 49 studies, they identified 44 unique apps, 12 of which were mobile apps. Most apps focused on education, social support, and self -management. However, a key limitation of existing apps was their emphasis on reducing negative emotions rather than enhancing positive emotions and resilience through strategies that support self-care and emotional fulfillment.

Existing mHealth interventions for caregivers have shown evidence of effectiveness, but they often target multiple domains beyond positive activities and have not focused on dementia caregivers. For example, Fuller-Tyszkiewicz et al. (2020) developed StressLess, a 5-week self-guided intervention for caregivers of individuals with and without ADRD. The intervention included psychoeducation on stress reduction, goal setting, mindfulness skills, well-being enhancement via cognitive restructuring, and encouragement to engage in pleasant activities. They were encouraged to complete one module per week, with flexibility to complete modules in any order and at any time. Participants completed EMAs up to four times a day assessing stress, coping, and mood, and they received app-based feedback based on their responses. Compared to an active control group that included EMAs and app-based feedback but no intervention components, participants in the intervention group reported reductions in stress and depression, as well as improved well-being. However, participants completed an average of only 2.55 of the 5 modules, suggesting that the app may have been burdensome and/or that participants may need prompts or additional support to sustain engagement. In addition, the sample included a broad range of caregivers, many of whom were mothers caring for disabled children, limiting conclusions specific to dementia caregivers.

Ferré-Grau et al. (2021) developed TIVA, a positive psychology-based mHealth app for caregivers of individuals with and without ADRD. Participants were instructed to use the app over a 28-day period. Compared with the control group, the intervention group reported improvements in several positive mental health outcomes at the three-month follow-up. Although promising, this intervention was not designed specifically for dementia caregivers and, like other caregiver-focused mHealth interventions, addressed a broader set of caregiver needs rather than focusing specifically on positive activity engagement.

Other apps developed for dementia caregivers have generally not emphasized positive activities. For instance, Yang et al. (2026) developed mWITH ME, an app with four modules: educational support, professional support, peer support, and health monitoring. Caregivers reported that the app helped reduce feelings of burden. Castillo et al. (2024) evaluated 16 mobile apps currently available for dementia caregivers and concluded that, although such apps are increasing in number, few have been evaluated in controlled studies and limited information is available about caregivers’ experiences using them. The authors suggested that existing apps may not fully meet the needs of dementia caregivers. The most common feature was provision of information on care management, while only a few apps included stress management activities. In a comparison of two specific apps, Dementia Talk and the Clear Dementia Care app, participants were asked to use an app for at least 1 h per week over 2 weeks. Participants reported a need for more self-care activities in both apps.

Overall, although many apps are available for caregivers, they often do not focus on improving positive mood, stress management, and self-care, features that both researchers and caregivers have identified as important gaps in existing caregiver-focused apps. To address these gaps, Choi developed an app-based mobile health intervention (Roadmap; R01HL146354: PI Choi), the Roadmap app (Maher et al., 2016; Cao et al., 2025), that provides eight positive activity modules for caregivers (Figure 2) (Runaas et al., 2017, 2018; Shin et al., 2018, 2020; Chaar et al., 2019; Rozwadowski et al., 2020). The modules were based on extensive qualitative interviews included in recently published studies (Runaas et al., 2017, 2018; Fauer et al., 2018; Shin et al., 2018, 2020; Chaar et al., 2019; Rozwadowski et al., 2020). This app has demonstrated feasibility, acceptability, and efficacy in caregivers across multiple populations (e.g., caregivers for patients with blood and marrow transplantation, caregivers of pediatric patients with cancer), as well as effectiveness for improving mental health and resilience (Cao et al., 2025). The app has yet to be assessed among dementia caregivers; thus, we will examine the feasibility and acceptability of using the Roadmap app among ADRD caregivers over 14 days, as well as explore preliminary data examining associations between engagement in app positive activities and caregiver mental health (primary), potential mechanisms of action (mood, appraisals of caregiving), and proxy reports of care recipient mental health (secondary). We address three research questions:

1. Is the Roadmap app feasible and acceptable to caregivers of people living with dementia?
2. Do ADRD caregivers report engaging in sufficient numbers of positive activities in the Roadmap app, and do they complete a sufficient number of nightly surveys for measuring mood and mental health?
3. Is there initial evidence suggesting that there are associations between Roadmap app positive activities and better mood, appraisals of caregiving, and mental health among caregivers? We also explore associations between positive activities and proxy reports of care recipient mental health.

**Figure 2:**
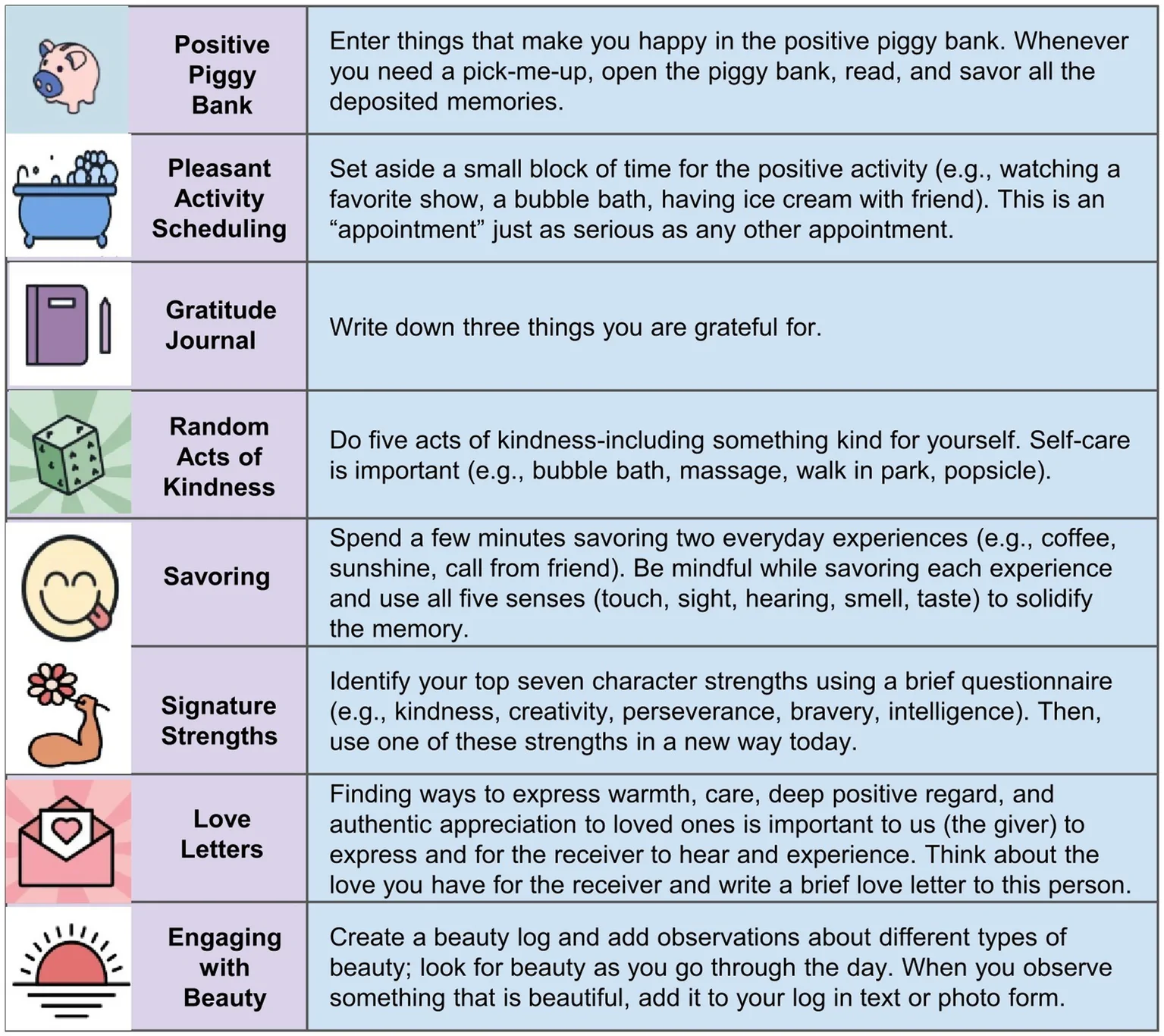
Roadmap positive activity app.

## Method

### Participants

A total of 20 ADRD caregivers participated in the We-Care (Well-Being Enhancement of Caregivers) study. These dyads were recruited from the SWEL-Care (Stress and Well-being in Everyday Lives of Caregivers, PI Birditt) study sample, which included a total of 223 dementia caregivers and their care recipients (Ng et al., 2025). Of the 46 caregivers who were invited to participate in the We-Care study, 6 indicated they were not interested, 1 was not eligible due to no longer living with the care recipient, 1 had incorrect contact information, and 18 were invited via email and phone but did not respond. Of the 20 caregivers who participated, 5 of their care recipients did not participate either because they were not eligible (1 recipient was nonverbal, 2 did not have a compatible phone) or declined to wear the Fitbit® (1 declined to participate and 1 consented but did not like wearing the Fitbit®). Thus, the total sample included 15 complete dyads (*n* = 30) and 5 caregivers without a participating care recipient. *Inclusion Criteria.* To be eligible for the We-Care study, caregivers must have completed baseline interviews, and the care recipients must have completed cognitive assessments (T-MoCA) during the SWEL-Care Study. Additionally, caregivers had to be: (1) living with the care recipient, (2) be the primary caregiver, and (3) have smartphones that could download the Fitbit and Roadmap app.

### Procedure

During recruitment, ADRD caregivers were told the purpose of the study is “to learn how a mobile health app that promotes well-being (called the Roadmap app) can improve the daily lives and well-being of dementia caregivers and their care partners”. Participants had a consent appointment and onboarding session, in which they learned how to use the Roadmap app and Fitbit®, before starting their 14-day daily assessment.

ADRD caregivers completed a 14-day intensive data collection. Caregivers received daily prompts at 9 a.m. and 6 p.m. to engage in a positive activity of their own choice that day. Each night, caregivers completed a mobile phone survey with questions regarding the well-being of the caregiver and care recipient. Both the caregiver and care recipient wore Fitbit® Inspire devices for 2 weeks (data from which we plan to analyze in a future manuscript). On the last day (Day 14), caregivers received a survey that also inquired about the ease of use of the Fitbit® and the Roadmap app.

Caregivers were paid $1 per nightly survey (total of $14 for 14 days), $2 per day for Fitbit® data (total of $28 for 14 days) and $25 for completion of the in-depth interview. Care recipients were paid $2 per day for Fitbit® data (total of $28 for 14 days). Both the caregiver and care recipients were able to keep the Fitbit® once the study was completed.

### Measures

#### Baseline interview

The baseline survey, conducted during SWEL-Care between February 2022 to June 2024, included sociodemographic data on the caregiver and care recipient. Caregivers also completed the 11-item Zarit Caregiver Burden scale (Bédard et al., 2001) that assesses feelings of stress and burden regarding the caregiving role. Response options included: Never = 0, Rarely = 1, Sometimes = 2, Quite frequently = 3, Nearly always = 4 and a sum score was calculated. Care recipients completed a Montreal Cognitive Assessment, specifically the telephone MoCA (T-MoCA) (Benge and Kiselica, 2021). The T-MoCA has adequate sensitivity and specificity for mild cognitive impairment (MCI). Scores from this measure were used to determine the severity of dementia, which includes: No impairment (26–30 = 0), Mild impairment (18–25 = 1), Moderate impairment (10–17 = 2), and Severe impairment (0–9 = 3) (Benge and Kiselica, 2021).

#### Nightly survey

##### Positive activities

Each night, caregivers were asked to select which of the 8 positive activities from the Roadmap app they engaged with during the day. We created a dichotomous score that reflected whether or not the caregiver reported engaging in a Roadmap positive activity, as well as a sum of positive activities reported each day.

##### Caregiver mental health

Caregiver mental health was evaluated with two-item custom short form from the Patient-Reported Outcomes Measurement Information System (PROMIS) Anxiety measure: (I found it hard to focus on anything other than my anxiety and My worries overwhelmed me), as well as two-item custom short form from the PROMIS Depression measure: I felt depressed and I felt hopeless. Response options include: Never = 1, Rarely = 2, Sometimes = 3, Often = 4, Always = 5 (Cella et al., 2007). The item scores were tabulated on a preformatted spreadsheet using anonymized participant identifiers; this was then uploaded onto the HealthMeasures scoring service, powered by the assessment center^1^ and the relevant *t*-scores were returned.

##### Caregiver mood

Caregiver mood (positive and negative affect) was evaluated using 3 positive affect items (content, calm, and happy; *ɑ* = 0.73) and 5 negative affect items (worried, irritated, lonely, stressed, sad; *ɑ* = 0.71), (Shaver et al., 1987; Watson et al., 1988). Response options include: Not at all = 1, A little = 2, Somewhat = 3, Quite a bit = 4, A great deal = 5. Average scores of these items were calculated to indicate positive and negative mood each day, with higher scores indicating greater positive and greater negative mood, respectively.

##### Caregiver appraisals

After reporting on the tasks they did for the care recipient, caregivers were asked to rate how stressful it was to provide help and how rewarding it was to provide help to the care recipient overall that day from 1 (not at all) to 5 (a great deal).

##### Proxy reports of care recipient’s mental health

Caregivers provided proxy reports of care recipient’s mental health with a four-item custom short form from the Neuro-QoL Anxiety measures (uneasy, nervous, worried, difficulty calming down) and a four-item custom short form from the Neuro-QoL Depression measure (depressed, nothing could cheer me up, unhappy, nothing was interesting) (Cella et al., 2012). Caregivers were instructed to respond as if they were the care recipient. Response options include: Never = 1, Rarely = 2, Sometimes = 3, Often = 4, Always = 5. Similarly, relevant *t*-scores were obtained from the HealthMeasures scoring service.

#### Final survey

##### Feasibility and acceptability

The final evening survey included a study-specific questionnaire (which was based on existing measures of feasibility and acceptability) was used to examine the feasibility and acceptability of the Roadmap app, the Fitbit®, and the study design (Vandelanotte and De Bourdeaudhuij, 2003; Haerens et al., 2007; Kratz et al., 2017; Carlozzi et al., 2022, 2025). Items regarding Roadmap app use were: (1) The instructions for the Roadmap app set up were easy to understand; (2) The Roadmap app was easy to set up; (3) The Roadmap app was easy to use; (4) I was confident using the Roadmap app; (5) I am confident that I was using the Roadmap app correctly (*a* = 0.89). Response options included Strongly Disagree (1), Disagree (2), Neither Agree nor Disagree (3), Agree (4) and Strongly Agree (5). A mean score was calculated for these items and as well as a percentage of agree/strongly agree across the items. Items regarding the use of Fitbit® included: (1) The instructions for the Fitbit®set up were easy to understand; (2) The Fitbit® was easy to set up; (3) The Fitbit® was easy to use; (4) The Fitbit® was comfortable to wear; (5) The Fitbit® data was easy to sync with my phone; (6) I was confident using the Fitbit® (*a* = 0.76). Response options included Strongly Disagree (1), Disagree (2), Neither Agree nor Disagree (3), Agree (4) and Strongly Agree (5). A mean score was calculated for these items as well as a percentage of agree/strongly agree across the items (Vandelanotte and De Bourdeaudhuij, 2003; Haerens et al., 2007; Kratz et al., 2017; Carlozzi et al., 2022, 2025). Items regarding the acceptability of the Nightly Surveys included: (1) The nightly survey questions were easy to understand. (2) The nightly survey questions were easy to answer. (3) Answering the nightly survey questions fit easily into my routine. (4) The number of nightly survey questions to answer on the app was reasonable. (*a* = 0.77). Response options included Strongly Disagree (1), Disagree (2), Neither Agree nor Disagree (3), Agree (4) and Strongly Agree (5). A mean score was calculated for these items as well as a percentage of agree/strongly agree across the items. We would consider there to be adequate support for feasibility and acceptability assuming ≥80% of the surveys were completed or ≥80% of the days included a positive activity, or ≥80% of respondents agreed or strongly agreed with the different components of the study (Carlozzi et al., 2022).

#### Analysis strategy

First, we calculated descriptive statistics to describe the sample, daily appraisals, mood, and mental health outcomes of the caregivers and the caregiver proxy reports of the mental health of the care recipient. Next, feasibility was examined with metrics including the proportion of days participants engaged in positive activities, the proportion of positive activities that fit into each of the eight Roadmap categories, and the proportion of days participants completed nightly surveys. Additional feasibility and acceptability data were examined by responses on the feasibility and acceptability survey that was completed on the last day of the study.

To examine associations between roadmap positive activities and mental health, we estimated two-level multilevel linear models (with days nested within participants) to examine whether there is preliminary evidence of associations between engaging in app-based positive activities and outcomes. These analyses are intentionally exploratory, as the primary aim of this study was to establish the feasibility and acceptability of the intervention. Consequently, the sample size was selected to address these aims rather than to provide adequate statistical power to detect associations estimated using multilevel models. Nonetheless, we fit these models to provide an initial understanding of the direction and magnitude of associations. Fitted models revealed substantial within-person variability (range: 43–74%) in all caregivers’ and care recipient outcomes and substantial within-person variability in positive activities (65%; see Supplementary Table 1). Time (assessed with a variable representing the day in the study and ranged from day 1 to 14), time-varying dichotomous indicator of engaging in any positive activities each day (within-person), and the percentage of days engaged in positive activities reported over the study period (between-person) were the predictors. *Post-hoc* analyses examined the sum of positive activities each day (within-person) and person-level average number of positive activities across the study period (between-person) as predictors. Caregivers’ outcomes included positive and negative mood, appraisals of caregiving (rewards and stress), and mental health (anxiety, depression) in six multilevel models. Caregiver proxy reports of care recipient outcomes included anxiety and depression as outcomes in two separate multilevel linear models. Across models, we adjusted for key covariates (caregivers’ race and burden score). We also examined caregivers’ age and gender, and care recipients’ T-MoCA scores as covariates but none of these were significantly associated with any of the outcome measures. We also estimated models without covariates to ensure the models were not overfit.

## Results

### Description of the study participants

See Table 1 for the sample demographics. The caregivers ranged in age from 43 to 82 (M = 61.15, SD = 11.69), and care recipients ranged in age from 38 to 92 (M = 74.20, SD = 11.59). A total of 60% were White and 40% Black; 75% were female. A total of 35% were adult children caring for parents, and 65% were spousal caregivers. A total of 75% caregivers had attained a college degree or above.

**Table 1:** Sample description (*N* = 20).

Demographics	Mean/%	SD	Min	Max
Caregiver age	61.15	11.69	43.00	82.00
Black	40%			
Caregiver female	75%			
Married	70%			
Higher education	75%			
Relationship to PLWD				
Spouse/partner	65%			
Adult child	35%			
Caregiver burden	21.25	10.84	7.00	44.00
Duration of help (years)	4.08	2.16	1.50	10.00
Care recipient age	74.20	11.59	38.00	92.00
Care recipient female	40%			
Care recipient T-MoCA score	13.25	8.23	0.00	29.00
T-MoCA dementia categories				
No impairment	10%			
Mild impairment	30%			
Moderate impairment	25%			
Severe impairment	35%			

Caregiver age is continuous in years. Black is coded as Black or African American = 1, White = 0. Caregiver female is coded as male = 0, female = 1. Higher education is a binary variable of holding a four-year college degree or higher (Yes = 1, No = 0). The duration of help is continuous in years. Care recipient age is continuous in years. Care recipient female is coded as male = 0, female = 1.

Caregivers reported an average score of 21.25 (SD = 10.84, range 7–44) on the caregiver burden scale. Based on the T-MoCA categorization, 10% of care recipients had no cognitive impairment (*n* = 2; 1 was diagnosed with dementia by a doctor and 1 received a score of 5 on the AD8, a 8-item informant interview assessing change in care recipient’s cognitive function), 30% had mild impairment, 25% had moderate impairment, and 35% had severe impairment (see Table 1). Caregivers reported relatively low levels of anxiety, depression, negative mood, and appraisals of stress, and moderate levels of positive mood and appraisals of caregiving as rewarding (Table 2). Caregivers also reported low levels of care recipient anxiety and depression.

**Table 2:** Descriptive statistics.

Daily variable	Mean/%	SD	Min	Max	*N*
Number of positive activities engaged in each day	1.66	1.66	0	6	265
Proportion of days engaged in positive activity	87.5	–	–	–	265
Caregiver daily anxiety (*t*-score)	49.19	7.67	43.50	74.50	265
Caregiver daily depression (*t*-score)	47.20	7.37	41.70	68.20	265
Caregiver daily positive mood	3.73	0.89	1	5	265
Caregiver daily negative mood	1.66	0.63	1	3.40	265
Appraisals of caregiving as stressful	1.88	0.91	1	5	263
Appraisals of caregiving as rewarding	3.48	1.22	1	5	263
Care recipient daily anxiety (*t*-score)	48.78	7.86	37.70	68.50	264
Care recipient daily depression (*t*-score)	46.97	7.28	37.20	64.50	264

*N* = Number of days. Caregiver daily anxiety had a possible raw score range of 2–10. Caregiver daily depression had a possible raw score range of 2–10. Care recipient daily anxiety had a possible raw score range of 4–20. Care recipient daily depression had a possible raw score range of 4–20.

*RQ1: Is the Roadmap app feasible and acceptable to caregivers of people living with dementia?*

There was generally support for the feasibility and acceptability of the different study elements (see Table 3) except for some issues with the Roadmap app. Findings met our prior specifications for the Fitbit® and for the nightly surveys, but fell slightly below what was expected for the Roadmap app. Specifically, overall acceptability of the Roadmap app fell just below our criterion (78.9%), with slightly lower than expected agreement with the following items: The instructions for the Roadmap app setup were easy to understand, The Roadmap app was easy to use, and I am confident that I was using the Roadmap app correctly. The lowest agreement among these items was 72% for confidence in using the app correctly. These items indicate areas to target for improvement in the instructions and in training participants how to use the app.

**Table 3:** Means and standard deviation scores on acceptability and feasibility items.

Item	Mean score (SD)	% (*n*)—agree/strongly agree
*Acceptability*		
Roadmap app	3.98 (0.82)	78.9% (18)^a^
The instructions for the Roadmap app setup were easy to understand.	3.89 (0.90)	77.8% (14)
The Roadmap app was easy to set up.	4.11 (0.83)	83.3% (15)
The Roadmap app was easy to use.	4.06 (1.00)	77.8% (14)
I was confident using the Roadmap app.	4.06 (1.11)	83.3% (15)
I am confident that I was using the Roadmap app correctly.	3.78 (1.06)	72.2% (13)
Fitbit®	4.12 (0.44)	89.8% (18)^a^
The instructions for the Fitbit® set up were easy to understand.	4.11 (0.58)	88.9% (16)
The Fitbit® was easy to set up.	3.94 (0.73)	83.3% (15)
The Fitbit® was easy to use.	4.00 (0.91)	88.9% (16)
The Fitbit® was comfortable to wear.	4.22 (0.65)	88.9% (16)
The Fitbit® data was easy to sync with my phone.	4.17 (0.51)	94.4% (17)
I was confident using the Fitbit®	4.28 (0.58)	94.4% (17)
Nightly surveys	4.32 (0.55)	91.7% (18)^a^
The nightly survey questions were easy to understand.	4.39 (0.61)	94.4% (17)
The nightly survey questions were easy to answer.	4.28 (0.75)	94.4% (17)
Answering the nightly survey questions fit easily into my routine.	4.22 (0.81)	88.9% (16)
The number of nightly survey questions to answer on the app was reasonable.	4.39 (0.70)	88.9% (16)
*Feasibility*	% (*n*)—total completion	
Nightly surveys	94.6% (265)	

a Average percentage agree/strongly agree across items per caregiver.

*RQ2: Do ADRD caregivers report engaging in sufficient numbers of positive activities in the Roadmap app, and do they complete a sufficient number of nightly surveys for measuring mood and mental health?*

As shown in Table 2, out of the two prompts received each day, caregivers completed 1.67 positive activities per day (SD = 0.75) and engaged in at least one app-based positive activity on 87% of days (range 59 to 100%). Caregivers completed an average of 13.25 nightly surveys (SD = 0.85; min = 12; max = 14) for a total of 265 days of survey data. Within the Roadmap app, caregivers preferred pleasant activity scheduling followed by savoring, gratitude journal, engaging with beauty, positive piggy bank, signature strengths, random acts of kindness, and love letters (see Supplementary Table 2). The response rate for the last survey, which asked respondents about the acceptability of the app, the nightly surveys and the Fitbit, was 90%.

*RQ3: Is there preliminary evidence that engaging Roadmap app positive activities is associated with better mood, appraisals of caregiving, and mental health among caregivers, and better proxy reports of mental health for care recipients?*

As shown in Tables 4, 5, on days in which caregivers engaged in at least one Roadmap positive activity (within-person), they reported lower depression (*b* = −2.30, SE = 0.94, *p* = 0.015), lower appraisals of caregiving as stressful (*b* = −0.39, SE = 0.16, *p* = 0.014), and higher positive mood (*b* = 0.30, SE = 0.12, *p* = 0.014). There were no significant associations between within-person positive activity engagement and caregiver anxiety, negative mood, or appraisals of caregiving as rewarding. There were no significant between-person effects of the percentage of days engaged in positive activities on any of the outcomes. Finally time in the study (day 1 through 14) was associated with higher positive mood (*b* = 0.04, SE = 0.01, *p* < 0.001), lower negative mood (*b* = −0.02, SE = 0.01, *p* = 0.002), higher appraisals of caregiving as rewarding (*b* = 0.03, SE = 0.01, *p* = 0.018), lower anxiety (*b* = −0.29, SE = 0.08, *p* < 0.001) and lower depression (*b* = −0.33, SE = 0.07, *p* < 0.001), but appraisals of caregiving as stressful did not significantly change over time (*b* = −0.02, SE = 0.01, *p* = 0.078). Due to the small sample, multilevel models were also estimated without covariates to examine whether the models were overestimated. There was no change in results after running models without covariates.

**Table 4:** Multilevel models examining caregiver mental health as a function of roadmap positive activities.

Variable	Daily anxiety (*t*-score)			Daily depression (*t*-score)		
	*b*	SE	95% CI	*b*	SE	95% CI
Intercept	57.09^***^	8.32	39.47, 74.71	50.04^***^	8.15	32.79, 67.30
Positive activity (within person)	−0.93	1.00	−2.90, 1.05	−2.30^*^	0.94	−4.15, −0.44
Positive activity (between person)	−11.68	9.13	−31.00, 7.64	−2.24	8.94	−21.16, 16.68
Time in study	−0.29^***^	0.08	−0.44, −0.15	−0.33^***^	0.07	−0.47, −0.19
Covariates						
Black	−2.29	2.48	−7.53, 2.96	−4.49	2.43	−9.64, 0.65
Caregiving burden	0.29^*^	0.11	0.05, 0.54	0.25^*^	0.11	0.02, 0.49
Observations	265			265		

^*^p < 0.05; ^**^p < 0.01; ^***^p < 0.001.

**Table 5:** Multilevel models examining mechanisms of action as a function of roadmap positive activities.

Variable	Positive mood			Negative mood			Helping PLWD stressful			Helping PLWD rewarding		
	*b*	SE	95% CI	*b*	SE	95% CI	*b*	SE	95% CI	*b*	SE	95% CI
Intercept	2.32^*^	1.02	0.16, 4.49	2.26^***^	0.52	1.15, 3.37	2.27^*^	0.82	0.55, 4.00	2.15	1.56	−1.15, 5.45
Positive activity (within person)	0.30^*^	0.12	0.06, 0.54	−0.16	0.09	−0.34, 0.01	−0.39^*^	0.16	−0.69, −0.08	−0.01	0.17	−0.34, 0.31
Positive activity (between person)	1.27	1.12	−1.10, 3.65	−0.85	0.58	−2.07, 0.37	−0.17	0.90	−2.07, 1.73	1.33	1.71	−2.29, 4.95
Time in Study	0.04^***^	0.01	0.02, 0.06	−0.02^**^	0.01	−0.04, −0.01	−0.02	0.01	−0.04, 0.00	0.03^*^	0.01	0.01, 0.06
Covariates												
Black	0.36	0.30	−0.29, 1.00	−0.35^*^	0.16	−0.68, −0.02	−0.29	0.24	−0.80, 0.22	0.11	0.46	−0.88, 1.09
Caregiving burden	−0.02	0.01	−0.05, 0.01	0.03^**^	0.01	0.01, 0.04	0.02	0.01	−0.01, 0.04	0.00	0.02	−0.05, 0.04
Observations	265			265			263			263		

^*^p < 0.05; ^**^p < 0.01; ^***^p < 0.001.

As shown in Table 6, multilevel models showed that time in the study was associated with lower proxy reports of anxiety (*b* = −0.27, SE = 0.08, *p* < 0.001) and lower proxy reports of depression among care recipients (*b* = −0.17, SE = 0.08, *p* = 0.033). There were no significant associations between caregiver engagement in at least one positive activity per day (within-person) or percentage of days the caregiver engaged in positive activities during the study period (between-person) and caregiver proxy reports of care recipient mental health.

**Table 6:** Multilevel models examining caregiver proxy reports of care recipient mental health outcomes as a function of Roadmap positive activities.

Variable	Care recipient daily anxiety (*t*-score)			Care recipient daily depression (*t*-score)		
	*b*	*SE*	95% CI	*b*	*SE*	95% CI
Intercept	46.51^***^	7.35	30.96, 62.06	49.10^***^	7.06	34.16, 64.04
Positive activity (within person)	−1.15	1.04	−3.19, 0.90	−0.50	1.03	−2.54, 1.54
Positive activity (between person)	−0.78	8.08	−17.84, 16.29	−6.40	7.76	−22.79, 10.00
Time in study	−0.27^***^	0.08	−0.43, −0.12	−0.17^*^	0.08	−0.32, −0.01
Covariates						
Black	−5.11^*^	2.18	−9.74, −0.48	−3.18	2.10	−7.62, 1.26
Caregiver burden	0.38^**^	0.10	0.17, 0.59	0.31^**^	0.10	0.10, 0.51
Observations	264			264		

^*^p < 0.05; ^**^p < 0.01; ^***^p < 0.001.

### *Post hoc* tests

We estimated models again with the total number of positive activities reported each day and the average number of positive activities reported over the study period as predictors. There were no within-person effects of the number of positive activities in a day predicting any of the outcomes. Caregivers who reported more positive activities on average (between-person) reported lower anxiety (*b* = −3.40, SE = 1.59, *p* = 0.048) and higher positive mood (*b* = 0.58, SE = 0.17, *p* = 0.003). Time in the study was associated with higher positive mood (*b* = 0.04, SE = 0.01, *p* < 0.001), lower negative mood (*b* = −0.02, SE = 0.01, *p* = 0.003), higher appraisals of caregiving as rewarding (*b* = 0.03, SE = 0.01, *p* = 0.016), lower anxiety (*b* = −0.29, SE = 0.08, *p* < 0.001), and lower depression (*b* = −0.32, SE = 0.07, *p* < 0.001), but appraisals of caregiving as stressful did not significantly change over time (*b* = −0.02, SE = 0.01, *p* = 0.116). Models estimated without covariates revealed that caregivers who reported more positive activities on average (between-person) reported lower negative affect (*b* = −0.30, SE = 0.13, *p* = 0.032).

## Discussion

The purpose of this study was to examine the feasibility and acceptability of the Roadmap positive activity app among dementia caregivers, as well as preliminary associations with caregiver mental health outcomes. Caregiver mental health was the primary outcome, and caregiver proxy reports of care recipient mental health were secondary outcomes. The Roadmap app is distinct from other caregiver-focused apps described in the literature because it focuses specifically on positive activities and on improving positive mood and mental health. This focus addresses a gap identified by both caregivers and researchers, who have noted the need for caregiver apps that include more self-care, positive mood, and stress-management content. Overall, findings support the feasibility and acceptability of the study design, while also identifying areas where the Roadmap app could be improved. The study also provides preliminary evidence that engagement with Roadmap positive activities may be associated with better caregiver mental health.

Overall, respondents reported engaging in the app’s positive activities more than 80% of the days, and they completed over 80% of the nightly surveys. These indicators show that using the app is feasible among dementia caregivers (Carlozzi et al., 2022). Further, these rates are consistent with or exceed other studies using the Roadmap app with other populations of caregivers (Cao et al., 2025).

Additional evidence supported the feasibility and acceptability of the broader study protocol. Participants generally reported that the Roadmap app, Fitbit®, and nightly surveys were easy to understand and use. However, three acceptability items related specifically to the Roadmap app did not meet our 80% criterion. These findings identify specific targets for improvement in future studies. For example, caregivers reported somewhat lower confidence in their ability to use the app correctly, with 72% endorsing this item. They also reported slightly lower agreement that the instructions were easy to understand and that the app itself was easy to use. These results suggest that caregivers may benefit from clearer instructions and more structured training on how to use the Roadmap app. Improving onboarding and instructions may also increase caregivers’ confidence in using the app correctly.

We also examined whether daily positive activity engagement and the total number of positive activities completed were associated with caregiver mental health outcomes and potential mechanisms of action (mood, appraisals of caregiving). Preliminary findings suggest that Roadmap app activities were associated with better caregiver mental health. On days when caregivers reported engaging at least one Roadmap positive activity, they reported lower depression, lower appraisals of caregiving as stressful, and higher positive mood. Across the study period, caregivers also reported improvements over time in mood, caregiving appraisals, and mental health. Although positive activities were not associated with caregiver proxy reports of care recipient mental health, caregivers did report improvements in care recipient mental health over time. Given the small sample size and the lack of randomization we recognize that these findings are preliminary and should be interpreted with caution. Observed changes over the 14-day study period may reflect repeated measurement, study participation effects, regression to the mean, natural day-to-day variation, or other unmeasured factors. Nevertheless, the findings are consistent with prior research showing that engagement in positive activities is associated with improved affect and mental well-being among ADRD caregivers and care recipients (Mausbach et al., 2017). For example, previous studies show that caregivers who engage in fewer pleasant activities report greater depression, greater negative affect, lower positive affect (Chattillion et al., 2013). Other intervention studies have also found that when compared to psychoeducation, dementia caregivers who also received pleasant activity scheduling reported decreased depression over 4 months (Lee et al., 2020) and other studies have found that interventions like LEAF (Life Enhancing Activities for Frontotemporal dementia family caregivers), which includes journaling about positive events and gratitude, mindfulness, and acts of kindness, was associated with increased positive affect, reduced negative affect, reduced caregiver burden, and stress over a 5- or 6-week period (Au et al., 2015). This study advances the field by demonstrating that a low-burden, scalable positive activity app may be useful and potentially beneficial for caregivers. However, a larger, fully powered randomized controlled trial with an appropriate comparison condition and follow-up assessment is needed to determine whether the Roadmap app improves caregiver and/or care recipient outcomes.

This study has several limitations that should be addressed in future research. The prompts for using the Roadmap app should be randomized to determine whether days with prompts are associated with better mental health and what time of day these prompts are most effective. The Roadmap app should eventually be compared with an attention control condition. An attention control is an active comparison condition designed to match the intervention group on nonspecific aspects of study participation, such as frequency of prompts, study contact, time spent using an app, and participant attention, while excluding the hypothesized active components of the intervention. Including such a condition in a future trial would help determine whether observed changes may be due to the Roadmap positive activity content, rather than to general app use, prompting, monitoring, or study participation effects.

The app needs to be tested among a larger sample of caregivers and over a longer period of time. In addition, the study should include a follow-up assessment (s) to understand how long the app is associated with better health. We also need to analyze the Fitbit® data to understand whether the app is associated with improvements in secondary physical health outcomes such as heart rate, activity, and sleep.

Overall, this study suggests that the Roadmap app is potentially feasible and acceptable for use among dementia caregivers. The app may help caregivers manage daily stress by encouraging engagement in positive activities. However, fully powered randomized controlled trials are needed to determine whether the app produces improvements in mental health among caregivers and their care recipients beyond the preliminary associations observed in this study. Future research should also examine potential mechanisms of action, including whether changes in mood and caregiving appraisals explain improvements in caregiver mental health. This line of work has the potential to support the development of an evidence-based, scalable, and usable app that addresses important unmet needs among dementia caregivers.

## Data Availability

The raw data supporting the conclusions of this article will be made available by the authors, without undue reservation.

## Footnotes

1
https://www.assessmentcenter.net/ac_scoringservice
