# Supplementary material for: Use of the Roadmap positive activity app in daily life among dementia caregivers: evidence of feasibility and acceptability
Source: Front Dement. 2026 Sep 23;5:1861809. doi: 10.3389/frdem.2026.1861809 (PMC13642210; doi:10.3389/frdem.2026.1861809)
Supplement: Supplementary file 1 [file Table_1.docx]

**Supplementary tables**

**Supplemental Table 1**

*Proportion of total variance due to between-person and within-person differences*

|  | Between-person differences | Within-person differences |
| --- | --- | --- |
| Caregivers |  |  |
| Daily anxiety (*t*-score) | 0.57 | 0.43 |
| Daily depression (*t*-score) | 0.57 | 0.43 |
| Daily positive mood | 0.52 | 0.48 |
| Daily negative mood | 0.49 | 0.51 |
| Helping PLWD stressful | 0.27 | 0.73 |
| Helping PLWD rewarding | 0.54 | 0.46 |
| Engaged in ANY positive activity | 0.26 | 0.74 |
| Number of positive activities engaged in | 0.35 | 0.65 |
| Proxy Reports of Care Recipients |  |  |
| Daily anxiety (*t*-score) | 0.56 | 0.44 |
| Daily depression (*t*-score) | 0.50 | 0.50 |

**Supplemental Table 2**

*Percent of days respondents engaged in each Roadmap positive activity*

|  | Days |  | |
| --- | --- | --- | --- |
| Positive Activity | *N* | Percent | |
| Pleasant Activity Scheduling | 98 | 37.0% |  |
| Savoring | 70 | 26.4% |  |
| Gratitude Journal | 67 | 25.3% |  |
| Engaging with Beauty | 57 | 21.5% |  |
| Positive Piggy Bank | 48 | 18.1% |  |
| Signature Strengths | 47 | 17.7% |  |
| Random Acts of Kindness | 44 | 16.6% |  |
| Love Letters | 10 | 3.8% |  |
| None | 33 | 12.5% |  |

Note. Participants could engage in more than one activity per day.
